# Nanoporous Metal–Organic Framework Thin Films Prepared Directly from Gaseous Precursors by Atomic and Molecular Layer Deposition: Implications for Microelectronics

**DOI:** 10.1021/acsanm.2c04934

**Published:** 2023-01-10

**Authors:** Jenna Multia, Dmitry E. Kravchenko, Víctor Rubio-Giménez, Anish Philip, Rob Ameloot, Maarit Karppinen

**Affiliations:** ‡ Department of Chemistry and Materials Science, Aalto University, Aalto FI-00076, Finland; § Centre for Membrane Separations, Adsorption, Catalysis and Spectroscopy, Katholieke Universiteit Leuven, Leuven 3001, Belgium

**Keywords:** atomic layer deposition, molecular layer deposition, porous thin films, krypton physisorption, methanol physisorption, metal−organic framework

## Abstract

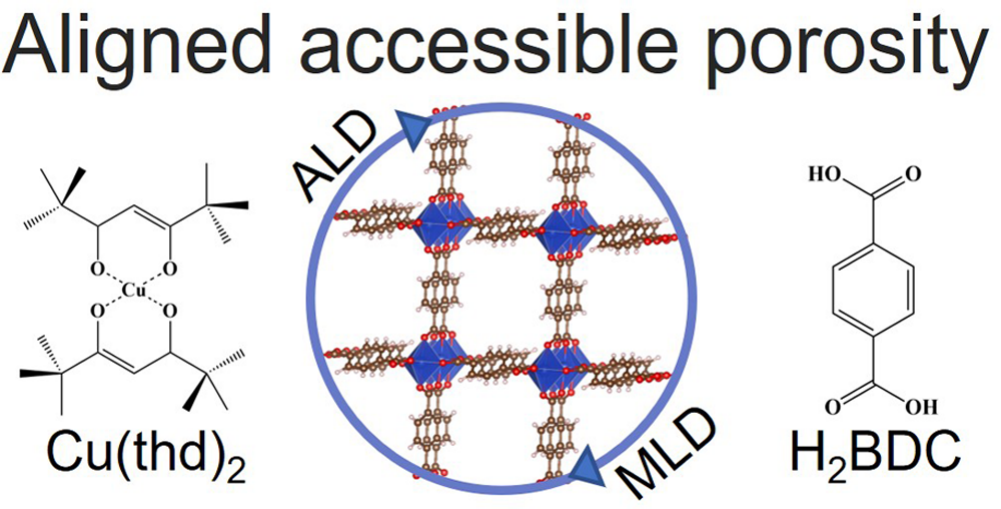

Atomic/molecular layer deposition (ALD/MLD) allows for the direct gas-phase synthesis of crystalline metal–organic framework (MOF) thin films. Here, we show for the first time using krypton and methanol physisorption measurements that ALD/MLD-fabricated copper 1,4-benzenedicarboxylate (Cu-BDC) ultrathin films possess accessible porosity matching that of the corresponding bulk MOF.

The landmark of the continuously expanding family of metal–organic frameworks (MOFs) is their highly porous structures.^1^ The open pores make these materials promising candidates for a variety of applications such as gas storage,^2^ separation,^3^ and catalysis.^4^ Solvothermal synthesis, together with pelletization and extrusion procedures, is typically utilized for these well-established uses.^5^ However, a more recent challenge is the integration of these porous MOF materials in microelectronics,^6^ which would require the development of industry-feasible nanoscale thin-film fabrication technologies.

In recent years, pioneering efforts have been made toward adopting cornerstone vapor-phase microfabrication techniques such as chemical vapor deposition (CVD) and atomic layer deposition (ALD) for the growth of MOF films.^7^ Hence, Stassen et al.^8^ developed the first MOF-CVD procedure to transform a metal oxide layer predeposited by ALD into a MOF structure via a solid–vapor reaction with organic linker vapor.

Alternatively, the combined atomic/molecular layer deposition (ALD/MLD) technique^9,10^ provides an elegant way to deposit metal–organic materials directly from gaseous precursors in a single process. Like ALD,^11^ the ALD/MLD technique is based on self-limiting gas-surface reactions of alternately supplied gaseous precursors, which enables thin-film uniformity and conformality as well as precise thickness control. So far, ALD/MLD processes have already been developed for more than a dozen in situ crystalline metal–organic materials, with both previously known and completely new crystal structures.^12−16^ Also, efforts have been made to crystallize the initially amorphous ALD/MLD-grown metal–organic films through various postdeposition treatments.^17−19^ This includes pioneering work by Ritala and co-workers for zinc 1,4-benzenedicarboxylate (Zn-BDC) films. Thus, a postdeposition treatment in a humidity-controlled chamber, followed by recrystallization with *N*,*N*-dimethylformamide in an autoclave, was required to obtain films of the cubic MOF-5 (also known as IRMOF-1) structure.^17^ Later, this work was followed by similar postdeposition crystallization efforts for IRMOF-8-structured zinc 2,6-naphthalenedicarboxylate films^18^ and UiO-66-structured zirconium 1,4-benzenedicarboxylate films.^19^

Some of the crystalline as-deposited ALD/MLD metal–organic films resemble the known MOF structures and thus are expected to be porous.^20−22^ However, their porous properties have not been experimentally verified. This can be mainly attributed to the experimental challenges of measuring the porosity on ultrathin (10–100 nm) films. The sensitivity of conventional volumetric N_2_ or Ar physisorption is too low to reliably characterize the minute amount of porous material on a typical substrate size (1–50 cm^2^). Recently, a number of alternative techniques have been employed to characterize the porous properties of thin films, such as positron annihilation lifetime spectroscopy, krypton physisorption (KrP),^23^ crystal microbalance (QCM) gravimetry,^13^ and ellipsometric porosimetry.^24^

In this paper, we for the first time show results from KrP porosity measurements for as-deposited crystalline ALD/MLD MOF films of copper 1,4-benzenedicarboxylate (Cu-BDC); the films were deposited following our previously reported ALD/MLD process based on Cu(thd)_2_ (thd = 2,2,6,6-tetramethyl-3,5-heptanedione) and 1,4-benzenedicarboxylic acid (H_2_BDC) precursors (Scheme 1).^21^ The porosity of our ALD/MLD Cu-BDC films was evaluated through KrP because of the accuracy of the technique for samples with extremely small surface areas (<1 m^2^), such as ultrathin films. In contrast to N_2_ and Ar, the sensitivity of KrP is significantly higher because of its much lower saturation pressure, thus reducing the number of molecules in the free space of the sample cell.^23,25^ In an earlier study, the porosity of Cu-BDC thin films was evaluated by QCM for samples prepared through a two-step CVD process consisting of a CuO/Cu precursor layer deposition and subsequent solid–vapor reaction of this layer with dicarboxylic acid linker vapor.^26^

**Scheme 1:**
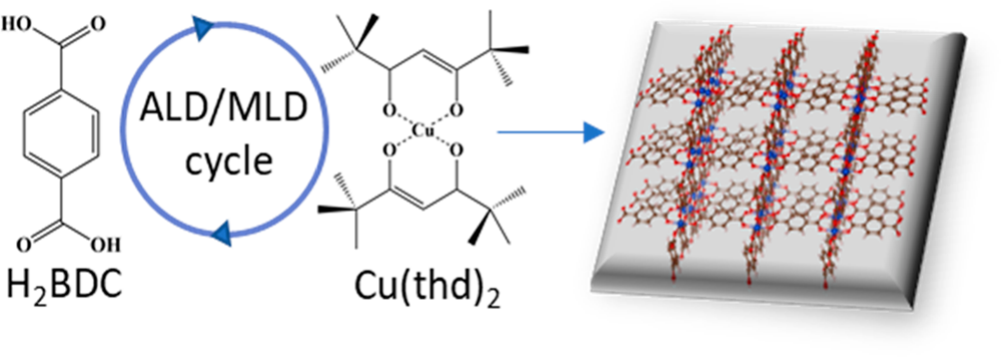
Schematic Illustration of a Crystalline ALD/MLD MOF Film of Cu-BDC Based on 1,4-Benzenedicarboxylic Acid and Cu(thd)_2_ Precursors Yielding Cu-BDC Films of the ZUBKEO^27^ Structure

The literature for solution-synthesized Cu-BDC materials in bulk form is already extensive (Table S1). The porous ZUBKEO (Figure S1) structure consists of dinuclear Cu^II^ moieties bridged with BDC linkers, forming two-dimensional sheets and one-dimensional pores.^27^ As demonstrated below, our ALD/MLD process yields phase-pure Cu-BDC films of the ZUBKEO structure.

The deposition of Cu-BDC films from Cu(thd)_2_ and H_2_BDC precursor powders is detailed in the Supporting Information (SI). All of the depositions were carried out at a chamber temperature ranging from 180 to 220 °C, with varying precursor/N_2_ purge pulse lengths depending on the type of substrate used. The samples meant for general characterization were deposited on silicon (Si) substrates with the following parameters: 5 s Cu(thd)_2_/2 s N_2_/10 s BDC/20 s N_2_. Samples for the KrP experiments were deposited on high-aspect-ratio (HAR) pillars substrates (further described in the SI) with 700 ALD/MLD cycles and with significantly longer pulse lengths to ensure conformal growth: 40 s Cu(thd)_2_/30 s N_2_/60 s BDC/80 s N_2_. Samples for QCM measurements were deposited straight on a SiO_2_ sensor substrate with 900 ALD/MLD cycles using the following precursor/purge protocol: 5 s Cu(thd)_2_/3 s N_2_/15 s BDC/20 s N_2_.

Next, we analyzed the crystallinity of our ALD/MLD Cu-BDC films. Because of their nanometric thicknesses, we measured synchrotron grazing-incidence X-ray diffraction (GIXRD). As shown in Figure 1a, the crystalline phase in our ALD/MLD Cu-BDC films could be readily assigned to the ZUBKEO Cu-BDC crystal structure, previously reported for powder Cu-BDC samples.^27^ A detailed inspection of the GIXRD reciprocal space maps revealed some degree of preferential orientation in the films, as denoted by the presence of small incomplete diffraction rings. As is visible in Figure 1b, their positions and intensities correspond to the ZUBKEO phase with a dominant (320) crystalline orientation. Thus, we assume that a significant percentage of the film’s crystallites are of that orientation, along with a minor fraction of other orientations and randomly oriented crystallites.

**Figure 1:**
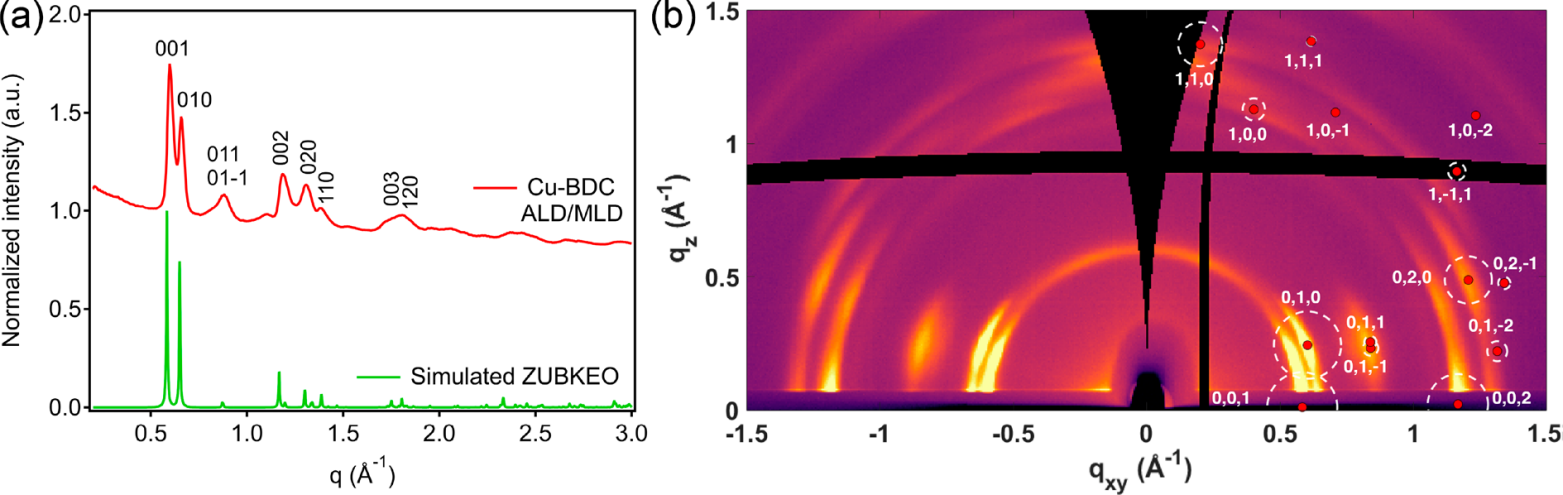
(a) Synchrotron GIXRD diffractogram for our ALD/MLD Cu-BDC thin film compared to the simulated pattern of reported crystal structure ZUBKEO.^27^ (b) Reciprocal space map of an ALD/MLD Cu-BDC thin film obtained from synchrotron GIXRD. The simulated Bragg peaks for a (320) crystalline orientation are overlaid on the positive *q*_*xy*_ side of the map. Red points at the center of circles give the expected positions of the diffraction peaks, and the areas inside the circles give the square of the structure factors, which are proportional to the expected intensities.

The film thickness determined through X-ray reflectivity (XRR) measurements was 44 nm for an ALD/MLD Cu-BDC film of 200 precursor pulse cycles (Figure 2a). The growth rate expressed as the so-called growth-per-cycle (GPC) value was thus 2.2 Å cycle^–1^ for samples deposited on Si wafer substrates. Densities were deduced from the critical angle θ_c_ in the XRR patterns using an equation detailed in the SI. The value obtained for our Cu-BDC film (1.5 g cm^–3^) is in reasonable agreement with the ideal density calculated for the ZUBKEO crystal structure (1.385 g cm^–3^).^27^ We ascribe the slightly higher density to a more strained structure in the ultrathin films versus the powder (Figure S2). The chemical bonding scheme expected for Cu-BDC was confirmed by Fourier transform infrared (FTIR; Figure 2b). The absence of the peak at 1720 cm^–1^ characteristic of the C=O stretch in a −COOH group indicates the completeness of the reaction between the precursors. Moreover, directly after the deposition, no features were seen around 3400 cm^–1^, which would indicate unintentionally adsorbed water. Finally, the bands around 1525 and 1390 cm^–1^ due to the asymmetric and symmetric vibrations of metal-coordinated carboxylate groups, respectively, confirm the expected bridging-type coordination of the carboxylate groups to the Cu^II^ atoms.^27−29^ Atomic force microscopy (AFM) images (Figures 2c and S3) show a smooth film surface over micrometric areas, composed of nanometric particles with a root-mean-square (RMS) roughness of 2.4 nm.

**Figure 2:**
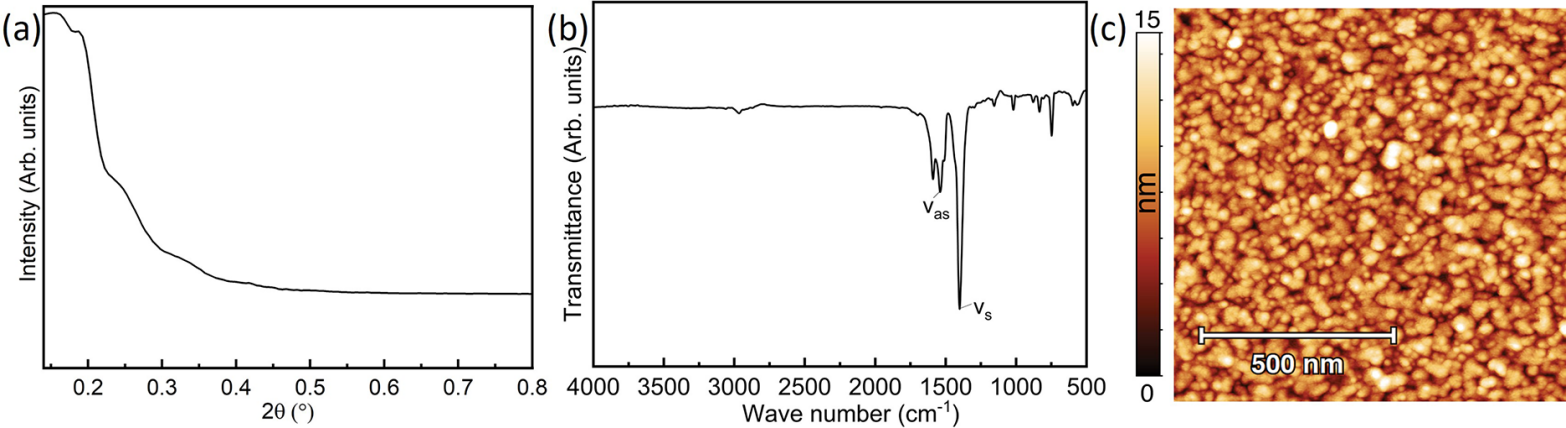
(a) XRR pattern, (b) FTIR spectrum, and (c) 1 × 1 μm^2^ AFM topography image for an ALD/MLD Cu-BDC film deposited on a Si substrate.

The porosity of our ALD/MLD Cu-BDC films was evaluated through KrP by depositing the Cu-BDC film (ca. 154 nm, extrapolated using the GPC value) on a substrate with HAR pillars to enhance the surface area and thus yield improved diffusion kinetics in comparison to films deposited on a flat Si substrate.

The Kr adsorption/desorption isotherms measured at 77 K for the sample after activation at 150 °C for 10 h compared with a blank HAR substrate are plotted in Figure 3a. According to the IUPAC classification,^30^ the ALD/MLD Cu-BDC film features a type I isotherm in the low-pressure region with a small H4-type hysteresis at *P*/*P*_0_ < 0.45, thus indicating a minor presence of mesoporosity likely originating from interparticle gaps. A specific surface area of 174 m^2^ cm^–3^ was obtained for the activated Cu-BDC film in the 0.001–0.07 *P*/*P*_0_ microporous region. Assuming that the film has a theoretical surface area of 1162 m^2^ cm^–3^ (calculated with *Zeo++* for the ZUBKEO structure^27,31^), this specific surface area corresponds to a 150-nm-thick film, in perfect agreement with the film thickness estimated using the GPC value. Alternatively, considering a thickness of exactly 154 nm, we obtain an experimental surface area of 1166 m^2^ cm^–3^, again matching the theoretical value for ZUBKEO Cu-BDC. In addition, according to the dominant (320) orientation revealed by synchrotron GIXRD, the pore channels are mostly aligned roughly normal to the surface (Figure S4), which should facilitate guest accessibility and contribute to the high Brunauer–Emmett–Teller (BET) area recorded.

**Figure 3:**
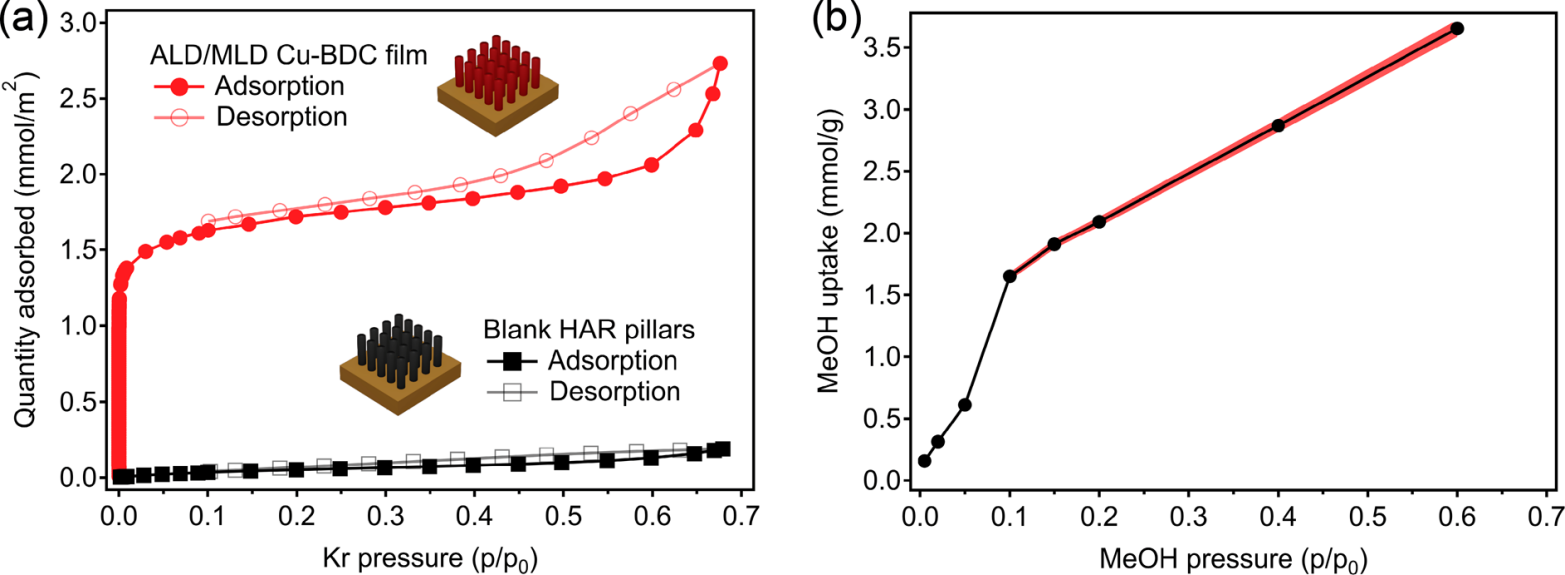
(a) KrP isotherms for the ALD/MLD Cu-BDC thin film and a blank HAR reference. (b) Methanol adsorption on a Cu-BDC-coated QCM sensor calculated for the 5th overtone. The red shaded area represents the 95% confidence interval based on the uncertainty in the MOF layer mass determination.

Methanol adsorption was measured by placing a Cu-BDC-coated sensor into a dedicated QCM cell, followed by exposure to methanol vapor of different concentrations. During the experiment, the sensor resonant frequencies (*f*_*n*_) and bandwidths (Γ_*n*_) for different overtones (*n* = 1, 3, 5, 7, 9, 11, and 13) were monitored. Because the change in the bandwidth (ΔΓ_*n*_) was much smaller than the resonant frequency shift (Δ*f*_*n*_) for all of the measured *n* (ΔΓ_*n*_/Δ*f*_*n*_ < 0.01), the Sauerbrey equation was applied to calculate the change in the layer’s mass corresponding to methanol adsorption.^32^ To determine the mass of the deposited Cu-BDC layer, it was first dissolved in piranha [1:3 (v/v) H_2_O_2_/H_2_SO_4_], and then *f*_*n*_ and Γ_*n*_ before and after were compared. Similarly, because ΔΓ_*n*_/Δ*f*_*n*_ < 0.01, the Sauerbrey equation was used to calculate the mass of the MOF layer. The specific methanol uptake calculated for *n* = 7 is shown in Figure 3b as an isotherm of shape similar to those of others of previously reported Cu-BDC thin films.^26^ The maximum uptake is in agreement with a rough estimation of 3.7 mmol g^–1^ based on the calculated ZUBKEO structure volume fraction (0.149 cm^3^ g^–1^) and density of liquid methanol (0.792 g cm^–3^).

In conclusion, we were able to directly produce crystalline and phase-pure thin films of a well-known Cu-BDC MOF via our ALD/MLD process. Moreover, synchrotron GIXRD measurements showed a preferential orientation of the film crystallites. The accessible porosity of these MOF thin films was demonstrated via KrP and methanol physisorption, with the total BET surface area and methanol uptake matching the theoretical values calculated for the ZUBKEO structure. Thus, we foresee that these positive results could serve as a strong motivation for further ALD/MLD process development for other promising porous MOF materials.

## Supporting Information Available

Additional experimental details and descriptions of the materials, substrates, characterization, and methods (PDF)
