# Supplementary material for: Nanoporous Metal–Organic Framework Thin Films Prepared Directly from Gaseous Precursors by Atomic and Molecular Layer Deposition: Implications for Microelectronics
Source: ACS Appl Nano Mater. 2023 Jan 10;6(2):827–31. doi: 10.1021/acsanm.2c04934 (PMC9887593; doi:10.1021/acsanm.2c04934)
Supplement: Supplementary file 1 — an2c04934_si_001.pdf [file an2c04934_si_001.pdf]

Supporting Information:

Nanoporous Metal-Organic Framework Thin Films Prepared Directly from Gaseous Precursors by Atomic and Molecular Layer Deposition: Implications for Microelectronics

Jenna Multia,[a, #] Dmitry E. Kravchenko,[b, #] Víctor Rubio-Giménez,[b, #] Anish Philip,[a] Rob Ameloot[b, *] and Maarit Karppinen[a, *]

[a] *Department of Chemistry and Materials Science, Aalto University, FI-00076 Aalto, Finland*
[b] *Centre for Membrane Separations, Adsorption, Catalysis and Spectroscopy (cMACS), KU Leuven, 3001 Leuven, Belgium*
* Corresponding author (email: maarit.karppinen@aalto.fi, rob.ameloot@kuleuven.be)
[#] *These authors contributed equally to this work*

Contents

Materials and substrates S1
Thin film deposition S1
Thin film characterization S2
Results S3
References S5

Materials and substrates

$Cu(thd)_2$ (thd = 2,2,6,6-tetramethyl-3,5-heptanedione) was synthesized following a previously reported procedure,[1] 4-benzenedicarboxylic acid (H_2BDC, >99.0%) was purchased from Tokyo Chemical Industry Co., Ltd. and used without further purification. Single-side polished p-type (100)-oriented silicon (Si, Okmetic Ltd.) substrates were used for most of the characterization. Samples meant for porosity characterization were deposited on high-aspect ratio (HAR) Silicon pillars as substrate. HAR substrates (staggered square array with pillar diameter of 2 μm, pillar height 50 μm and inter pillar distance of 2 μm) were produced by means of reactive ion etching (Bosch process) using 600 nm silicon oxide patterned by photolithography as the hard mask. For methanol porosimetry, films were deposited straight on SiO_2-coated 5 MHz AT-cut quartz crystal microbalance sensors (AWSensors).

Thin film deposition

The Cu-BDC films were deposited from $Cu(thd)_2$ and H_2BDC precursor powders. The depositions were carried out in a commercial flow-type hot-wall ALD reactor (F-120 by ASM Microchemistry Ltd.), in which the solid precursors were placed in open glass crucibles and heated at 103 °C ($Cu(thd)_2$) or 185 °C (H_2BDC) for sublimation. The reactor pressure was ~3 mbar with nitrogen (99.999%; Parker HPN 5000 N_2 generator) used as both the purging and carrier gases.

Thin film characterization

Synchrotron Grazing incidence X-ray diffraction (GIXRD). The crystallinity of the ALD/MLD Cu-BDC films was measured by GIXRD at beamline XRD1 of the Elettra synchrotron (wavelength: 1.4 Å, incidence angle: 0.8°). The diffraction pattern of a ca. 100-nm thick Cu-BDC film onto Si was obtained from 2D detector pixel images using the GIDVis software package.[2] The film thickness was determined through X-ray reflectivity measurements (XRR; X'Pert Pro, PANalytical; CuK_α; time per step 6 s).

X-ray reflectivity (XRR). Measurements were performed on a Malvern PANalytical Empyrean diffractometer using a Cu anode (Cu Kα1 = 1.5406 Å; Cu Kα2 = 1.5444 Å) operating at 40 mA and 45 kV with a step size of 0.005 ° and a time per step of 6 s. X'Pert Pro software (Malvern PANalytical) was used to fit the data. Densities were deduced from the critical angle θ_c in the XRR patterns, using equation $\rho_e = (\theta_c^2\pi)/(\lambda^2 r_e)$, where ρ_e is the mean electron density, λ is the X-ray wavelength, and r_e is the classical electron radius. By assuming the elemental composition as pure copper-dicarboxylate ($C_8H_4CuO_4$), the mass density was estimated from $\rho_m = (\rho_e A)/(N_A Z)$, where A is the average molar mass, N_A is the Avogadro constant, and Z is the average atomic number.

Fourier transform infrared spectroscopy (FTIR). Measurements were performed in transmission mode in the range 400–4000 cm^{-1} on a Bruker alpha II infrared Spectrometer. The spectrum represents the average of 24 scans at 4 cm^{-1} resolution after subtracting the blank Si reference spectrum.

Krypton physisorption (KrP). The specific surface area of the sample (in $m^2\ m^{-2}$) was calculated from the Kr adsorption isotherms measured at 77 K (Micromeritics 3Flex 3500 gas physisorption instrument). The sample was degassed prior to measurement at 150 °C under vacuum (10^{-2} mbar) for 10 h to ensure complete activation. Gas uptake equilibria were measured as described elsewhere.[3] To calculate the specific surface area expressed per unit of substrate area, the total surface area of the MOF (0.51 m^2) was divided by the geometrical area of the HAR pillars. The surface area was calculated by applying the BET method in the region 0.001-0.07 P/P_0 satisfying the Rouquerol criteria.[4] A Kr adsorptive cross-sectional area of 0.210 nm^2 was assumed for the BET calculations. The BET surface area of the sample was corrected by subtracting the area of a blank measurement (uncoated HAR pillars). The theoretical surface area of the ZUBKEO structure was calculated by Monte Carlo sampling using Zeo++.[5] The Kr hard sphere radius of 1.8 Å[6] at the triple point was used as an approximation for the Kr probe radius at 77 K.

Quartz crystal microbalance (QCM) porosimetry. Methanol adsorption was measured by placing a Cu-BDC-coated sensor into a dedicated QCM cell (X4 AWSensors), followed by exposure to methanol vapor of different concentrations. Before the measurement, the sample was mildly activated in-situ at 40 °C under a nitrogen flow. Methanol vapor was then generated by an in-house built set-up consisting of a mass-flow controller (MFC) feeding nitrogen to a methanol-filled bubbler and another MFC controlling the diluting nitrogen flow. The bubbler was immersed in a thermostatic bath to precisely control the saturated vapor pressure. Both the bath and the QCM cell were kept at 23.0 °C. During the experiment, the sensor resonant frequencies (f_n) and bandwidths (Γ_n) for different overtones (n = 1, 3, 5, 7, 9, 11, 13) were monitored. As the change in the bandwidth ($\Delta\Gamma_n$) was much smaller than the resonant frequency shift (Δf_n) for all the measured n ($\Delta\Gamma_n/\Delta f_n < 0.01$), the Sauerbrey equation was applied to calculate the change in the layer's mass corresponding to the methanol adsorption.[7] To determine the mass of the deposited Cu-BDC layer, it was first dissolved in Piranha (H_2O_2:H_2SO_4 1:3 v/v) and then the f_n and Γn of the same bare sensor were compared to those of the sensor after Cu-BDC deposition. Similarly, as $\Delta\Gamma_n/\Delta f_n < 0.01$, the Sauerbrey equation was used to calculate the mass of the MOF layer.

Atomic force microscopy (AFM). Samples were scanned under ambient conditions using a Bruker Multimode 8 AFM in tapping mode. Silicon tips with a natural resonance frequency of 300 kHz and with an equivalent constant force of

26 N·m^{-1} (AC160TS-R3, Olympus Corporation) were used. The scan rate was adjusted during the scanning of each image (0.1-1 Hz, 512 samples/line). Data processing and analysis were carried out with Gwyddion software.[8]

Results

Table S1. Previously reported Cu-BDC crystalline structures, detailing lattice parameters and data from CCDC database, Cambridge, UK. Two dense (JIBFUV and KAKSUL) and one porous (ZUBKEO) Cu-BDC structures have been identified so far. The JIBFUV structure consists of Cu-octahedral chains bridged with water molecules through hydrogen bonding, while the KAKSUL structure is a three-dimensional non-porous coordination polymer consisting of $Cu(OH)_2$ layers intercalated by terephthalate anions. Finally, the porous ZUBKEO (Figure S1) structure consists of dinuclear Cu^{II} moieties bridged with BDC linkers forming two-dimensional sheets and one-dimensional pores. A similar phase (PUYREH) was originally reported with DMF occupying the pores and coordinating to the Cu^{II} dimers.

CCDC identifier	CCDC code	Space group	a (Å)	b (Å)	c (Å)	**α** (°)	**β** (°)	γ (°)	Reference
JIBFUV	1185709	P b c n	6.869(3)	22.985(11)	6.298(3)	90	90	90	9
KAKSUL	1192591	P-1	10.1423(4)	6.3388(2)	3.4841(1)	99.170(2)	96.567(2)	98.760(3)	10
ZUBKEO	1056985	P-1	5.2502	9.66899	10.76792	90.291	91.059	92.413	11
PUYREH	687690	C 2/m	11.4143(3)	14.2687(4)	7.7800(2)	90	108.119(1)	90	12

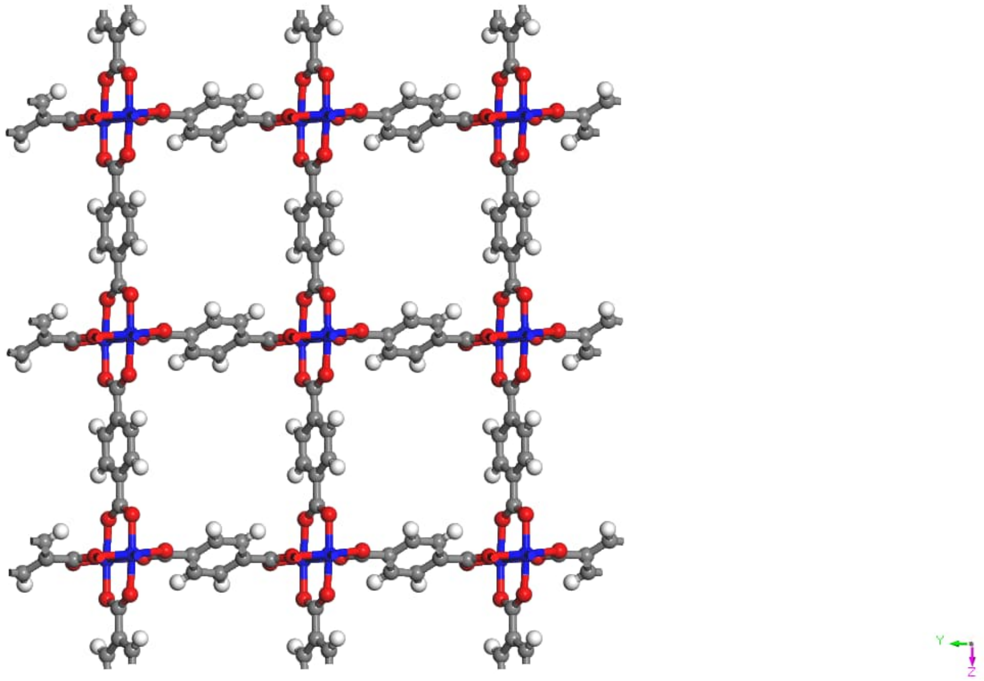

Figure S1. Structure of the ZUBKEO phase (CCDC 1056985) for Cu-BDC viewed along the a axis. Cu, O, C, and H atoms are colored blue, red, grey and white, respectively.

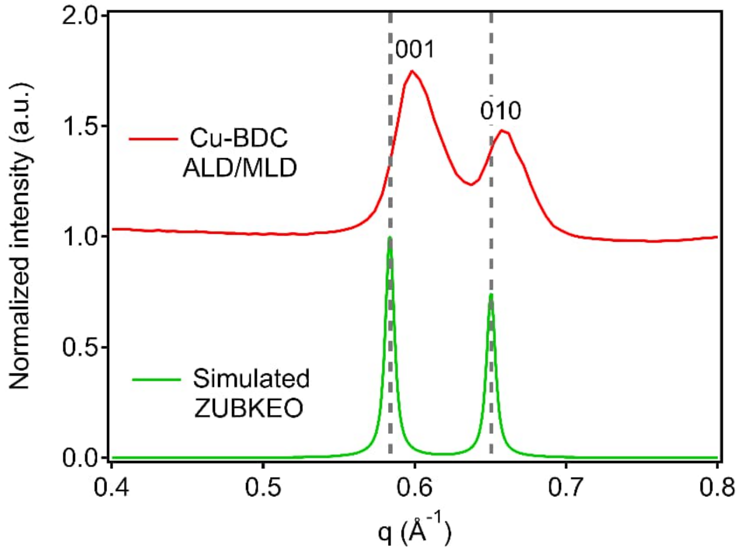

Figure S2. Zoom-in of the synchrotron GIXRD diffractogram of our ALD/MLD Cu-BDC thin film and the simulated ZUBKEO pattern. The experimental 001 and 010 peaks are shifted to higher q values, thus indicating a strained crystal cell.

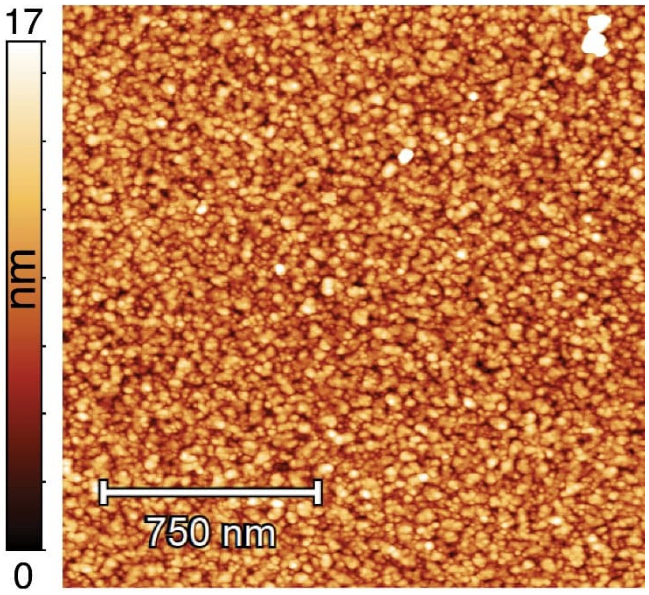

Figure S3. 2x2 µm^2 AFM topography image of an ALD/MLD Cu-BDC thin film deposited on Si.

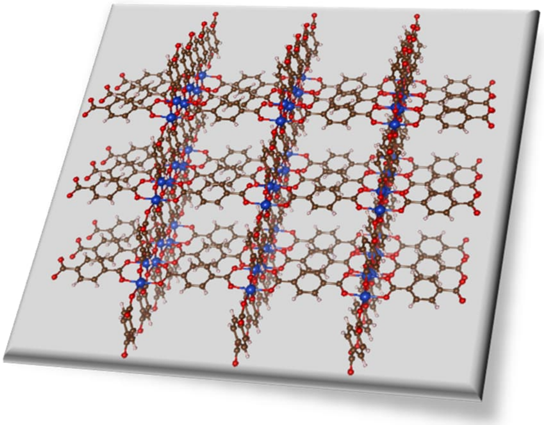

Figure S4. View of the crystalline structure of the Cu-BDC ZUBKEO phase, drawn with VESTA[13] showing the dominant (320) orientation revealed by synchrotron GIXRD. The pore channels are mostly aligned roughly normal to the surface of the substrate. Cu, O, C, and H atoms are colored blue, red, brown, and white, respectively.

References

(1) Hammond, G. S.; Nonhebel, D. C.; Wu, C. H. S. Chelates of β-Diketones. V. Preparation and Properties of Chelates Containing Sterically Hindered Ligands. *Inorg. Chem.* 1963, *2* (1), 73–76. https://doi.org/10.1021/IC50005A021/ASSET/IC50005A021.FP.PNG_V03.

(2) Schrode, B.; Pachmajer, S.; Dohr, M.; Röthel, C.; Domke, J.; Fritz, T.; Resel, R.; Werzer, O. GIDVis : A Comprehensive Software Tool for Geometry-Independent Grazing-Incidence X-Ray Diffraction Data Analysis and Pole-Figure Calculations. *J. Appl. Crystallogr.* 2019, *52* (3), 683–689. https://doi.org/10.1107/S1600576719004485.

(3) Stassen, I.; Styles, M.; Grenci, G.; Gorp, H. Van; Vanderlinden, W.; Feyter, S. De; Falcaro, P.; Vos, D. De; Vereecken, P.; Ameloot, R. Chemical Vapour Deposition of Zeolitic Imidazolate Framework Thin Films. *Nat. Mater.* 2016, *15* (3), 304–310. https://doi.org/10.1038/nmat4509.

(4) Rouquerol, J.; Llewellyn, P.; Rouquerol, F. Is the Bet Equation Applicable to Microporous Adsorbents? In *Studies in Surface Science and Catalysis*; Elsevier Inc., 2007; Vol. 160, pp 49–56. https://doi.org/10.1016/S0167-2991(07)80008-5.

(5) Willems, T. F.; Rycroft, C. H.; Kazi, M.; Meza, J. C.; Haranczyk, M. Algorithms and Tools for High-Throughput Geometry-Based Analysis of Crystalline Porous Materials. *Microporous Mesoporous Mater.* 2012, *149* (1), 134–141. https://doi.org/10.1016/j.micromeso.2011.08.020.

(6) Nezbeda, I.; Aim, K. Perturbed Hard-Sphere Equations of State of Real Fluids. II. Effective Hard-Sphere Diameters and Residual Properties. *Fluid Phase Equilib.* 1984, *17* (1), 1–18. https://doi.org/10.1016/0378-3812(84)80010-2.

(7) Reviakine, I.; Johannsmann, D.; Richter, R. P. Hearing What You Cannot See and Visualizing What You Hear: Interpreting Quartz Crystal Microbalance Data from Solvated Interfaces. *Anal. Chem.* 2011, *83* (23), 8838–8848. https://doi.org/10.1021/ac201778h.

(8) Nečas, D.; Klapetek, P. Gwyddion: An Open-Source Software for SPM Data Analysis. *Open Phys.* 2012, *10* (1), 181–188. https://doi.org/10.2478/s11534-011-0096-2.

(9) Cueto, S.; Gramlich, V.; Petter, W.; Rys, F. S.; Rys, P. Structure of Copper(II) Terephthalate Trihydrate. *Acta Crystallogr. Sect. C Cryst. Struct. Commun.* 1991, *47* (1), 75–78. https://doi.org/10.1107/S0108270190006345.

(10) Abdelouhab, S.; François, M.; Elkaim, E.; Rabu, P. Ab Initio Crystal Structure of Copper(II) Hydroxy-Terephthalate by Synchrotron Powder Diffraction and Magnetic Properties. *Solid State Sci.* 2005, *7* (2), 227–232. https://doi.org/10.1016/j.solidstatesciences.2004.10.020.

(11) Carson, C. G.; Brunnello, G.; Lee, S. G.; Jang, S. S.; Gerhardt, R. A.; Tannenbaum, R. Structure Solution from Powder Diffraction of Copper 1,4-Benzenedicarboxylate. *Eur. J. Inorg. Chem.* 2014, *2014* (12), 2140–2145. https://doi.org/10.1002/ejic.201301543.

(12) Carson, C. G.; Hardcastle, K.; Schwartz, J.; Liu, X.; Hoffmann, C.; Gerhardt, R. A.; Tannenbaum, R. Synthesis and Structure Characterization of Copper Terephthalate Metal-Organic Frameworks. *Eur. J. Inorg. Chem.* 2009,

2009 (16), 2338–2343. https://doi.org/10.1002/ejic.200801224.
(13) Momma, K.; Izumi, F. VESTA 3 for Three-Dimensional Visualization of Crystal, Volumetric and Morphology Data. *J. Appl. Crystallogr.* 2011, *44* (6), 1272–1276. https://doi.org/10.1107/S0021889811038970.
